# Novel combined pharmacological strategy to alleviate acute phase response following zoledronic acid treatment

**DOI:** 10.1007/s11657-024-01452-w

**Published:** 2024-10-15

**Authors:** Chung-Hwan Chen, En Kee Yeap, Chia-Hao Hsu, Yen-Mou Lu, Tsung-Lin Cheng, Tien-Ching Lee, Cheng-Jung Ho, Jhong-You Li, Hsin-Yi Shen, Hsuan-Ti Huang, Cheng-Chang Lu, Sung-Yen Lin

**Affiliations:** 1 Orthopaedic Research Center, Kaohsiung Medical University, Kaohsiung, Taiwan; 2 Division of Adult Reconstruction Surgery, Department of Orthopedics, Kaohsiung Medical University Hospital, Kaohsiung Medical University, Kaohsiung, Taiwan; 3 Department of Orthopedics, College of Medicine, Kaohsiung Medical University, Kaohsiung, Taiwan; 4 Department of Orthopedics, Kaohsiung Municipal Ta-Tung Hospital, Kaohsiung Medical University, Kaohsiung, Taiwan; 5 Regenerative Medicine and Cell Therapy Research Center, Kaohsiung Medical University, Kaohsiung, Taiwan; 6 College of Engineering, Graduate Institute of Materials Engineering, National Pingtung University of Science and Technology, Pingtung, Taiwan; 7 Department of Healthcare Administration and Medical Informatics, Kaohsiung Medical University, Kaohsiung, Taiwan; 8 Institute of Medical Science and Technology, National Sun Yat-Sen University, Kaohsiung, Taiwan; 9 Graduate Institute of Animal Vaccine Technology, College of Veterinary Medicine, National Pingtung University of Science and Technology, Pingtung, Taiwan; 10 Program in Biomedical Engineering, College of Medicine, Kaohsiung Medical University, Kaohsiung, 80708 Taiwan; 11 Department of Surgery, Kaohsiung Medical University Hospital, Kaohsiung, Taiwan; 12 Departments of Physiology, Collage of Medicine, Kaohsiung Medical University, Kaohsiung, Taiwan; 13 Department of Orthopedics, Kaohsiung Municipal Hsiao-Kang Hospital, Kaohsiung Medical University, Kaohsiung, Taiwan; 14 School of Post-Baccalaureate Medicine, College of Medicine, Kaohsiung Medical University, Kaohsiung, 80708 Taiwan; 15 Department of Orthopedics, Kaohsiung Medical University Gangshan Hospital, Kaohsiung, 820 Taiwan

**Keywords:** Acute phase response, Osteoporosis, Uveitis, Zoledronic acid

## Abstract

***Summary*:**

**Brief rationale:** Zoledronic acid treatment against osteoporosis is limited by APR. **Main result:** Combination therapy (hydrocortisone plus non-steroidal anti-inflammatory drugs, acetaminophen, and prednisolone) reduced intolerable APR levels and provided complete symptom relief in most patients. **Significance of the paper:** Combination therapy can enhance patient outcomes in osteoporosis management.

**Purpose:**

Osteoporosis is a common condition associated with high morbidity rates, often requiring treatment with bisphosphonates such as zoledronic acid. However, the persistence to zoledronic acid infusion is commonly limited by acute phase response (APR). This retrospective study aimed to evaluate the efficacy of a novel combination therapy in preventing APR symptoms.

**Methods:**

A retrospective case–control study was conducted on 931 patients who received their first zoledronic acid infusion between 2011 and 2021. We evaluated the efficacy of combination therapy comprising a single dose of hydrocortisone prior to the infusion and a 3-d oral regimen of non-steroidal anti-inflammatory drugs, acetaminophen, and prednisolone following the infusion. Patients were divided into protocol (receiving combination therapy) and control groups (without treatment). Baseline characteristics, APR incidence, and the efficacy of symptom control were compared between groups using Fisher’s exact test and Student’s *t*-test.

**Results:**

There was no difference in APR incidence between the protocol (*n* = 507) and control group (*n* = 407; *p* = 0.1442). However, the protocol group exhibited lower intolerable APR levels (3.72% vs. 16.71%; *p* < 0.0001) and complete symptom relief in 96.28% of cases.

**Conclusion:**

The combination therapy protocol effectively reduced intolerable APR and relieved symptoms in most patients following zoledronic acid infusion. This study highlights the importance of proactive management strategies for APR and emphasizes the potential of combination therapy in alleviating APR symptoms and reducing the occurrence of severe APR in patients undergoing osteoporosis management.

## Introduction

Osteoporosis affects ~ 9 million people worldwide [1]. Its global prevalence is increasing due to the aging population, leading to increased osteoporosis-related fractures. These fractures not only diminish the individual’s functional capacity and quality of life but also necessitate repeated hospitalizations and longer hospital stays, indirectly contributing to elevated mortality rates over the general population [2]. Various anti-osteoporotic agents have been developed, among which bisphosphonates are the primary clinical agents due to their notable effectiveness in reducing hip and vertebral fractures as well as their cost-effectiveness [3–5].

Zoledronic acid, a third-generation bisphosphonate, has been shown to effectively increase bone mineral density (BMD), decrease bone resorption marker levels, and reduce the incidence of new fractures across numerous clinical trials and studies [6–8]. Requiring only a single yearly dose, zoledronic acid offers high compliance and convenience, making it a preferred option for many patients [9]. Additionally, while discontinuation of denosumab can lead to a rebound phenomenon, causing a significant decline in BMD and an increased risk of multiple vertebral fractures, such rebound effects are not observed with zoledronic acid [10, 11]. Given that discontinuation of anti-osteoporosis treatments is common in clinical practice, zoledronic acid may represent a safer option due to its more stable profile regarding BMD after cessation. However, despite its benefits in osteoporosis management, the discomfort associated with acute phase response (APR) following zoledronic acid infusion has limited treatment adherence in clinical practice [12].

APR may occur in up to 42.4% of patients following their initial infusion and presents as a cluster of nonspecific symptoms, including fever, bone pain, myalgia, general weakness, and gastrointestinal discomfort [13]. The symptoms typically peak between 24 to 36 h post-infusion and are usually diminished after 2–3 d [13, 14]. Although the mechanism of APR remains debated, gamma/delta T cells (γ/δ T cell) may play a major role [15]. Various risk factors for APR have been identified, including non-Japanese Asian ethnicity, younger age, and non-steroidal anti-inflammatory drug (NSAID) usage [13]. Lower serum 25(OH)D levels have also been recognized as a predisposing factor [16]. Given that the Asian population tends to have lower serum 25(OH)D levels than Caucasians, the prevention of APR is especially challenging in this population [17, 18].

Numerous studies have investigated strategies for preventing and treating APR following zoledronic acid infusion. However, most studies have focused on single drug interventions. A single dose of dexamethasone or pretreatment with fluvastatin has been ineffective in controlling APR [19, 20]. Conversely, pre-treatment with NSAIDs before zoledronic acid administration effectively reduced APR incidence [21]. Notably, previous studies on the effect of NSAIDs did not solely include patients who were bisphosphonate-naïve, despite APR severity and incidence decreasing with repeated exposure [21]. Acetaminophen administration before and after zoledronic acid infusion has also been effective in managing APR; however, studies assessing the effectiveness of acetaminophen have predominantly focused on specific symptoms such as fever, headache, myalgia, or arthralgia, overlooking the varied spectrum of APR symptoms [19].

### Purpose

Zoledronic acid remains an important conventional therapeutic agent in osteoporosis management, particularly given the potential for managing side effects with other medication. In this study, we introduce a novel combination therapy—a regimen incorporating both prophylactic and rescue medications—to mitigate APR symptoms subsequent to zoledronic acid administration. The aim of this retrospective study was to evaluate the effectiveness of this prophylactic protocol in managing APR symptoms following the initial zoledronic acid infusion.

## Methods

This retrospective case–control study aimed to validate the efficacy of our innovative combinatorial therapy protocol in mitigating APR symptoms. We compared the APR incidence between two groups: one treated with our prophylactic protocol (protocol group) and another receiving no prophylactic treatment following zoledronic acid administration (control group). The combination protocol is outlined in Table 1.

**Table 1:** Protocol of combination therapy following zoledronic acid

**A**	**Premedication**
1	Hydrocortisone, 100 mg, i.v. bolus
2	A 250 mL pack of normal saline was administered via i.v. drip at 50 mL/hr before zoledronic acid infusion
**B**	**Zoledronic acid infusion**
1	Zoledronic acid, combined with 200 mL normal saline, was administered via i.v. drip over 1 h
**C**	**Rescue medications**
1	Prednisolone, 5 mg, 1 Tab TID for 3 d
2	Celecoxib, 1 Tab QD for 3 d
3	Acetaminophen, 1 Tab TID for 3 d

Patients who underwent their initial zoledronic acid treatment between January 2011 and June 2021 were included in this study. Baseline characteristics such as age, sex, BMD, and presence of fractures were obtained through retrospective chart review. Osteoporosis diagnosis was based on the World Health Organization's operational disease definition, which includes a T-score of ≤ –2.5 compared to young healthy women or the presence of fragility fractures. Patients not meeting these criteria were excluded from the study. Previous administration of osteoporotic agents other than zoledronic acid was not an exclusion criterion. The protocol group comprised patients who received the prophylactic protocol concurrently with zoledronic acid infusion, while the control group comprised those who did not receive any preventive regimen. Adverse events occurring after zoledronic acid infusion were recorded during outpatient department visits. Symptoms were compared to those identified in a previous study and matched symptoms were classified as APR [7].

In the control group, the occurrence of an APR was recorded only if patients returned for additional medication following zoledronic acid injection, indicating that only severe APRs were included in the analysis. In the protocol group, the effectiveness of the prevention protocol was assessed and classified into three subgroups: 1. APR prevented: All APR symptoms were effectively managed, resulting in either no symptoms or only minimal symptoms that did not significantly impact the patient. 2. Mild APR: APR symptoms were present but mild and tolerable, not requiring significant intervention. 3. Severe APR: APR symptoms were severe, requiring additional treatment or rest for management.

### Statistical analysis

Statistical analysis was conducted using GraphPad Prism Version 8.3.0 (GraphPad Software, San Diego, CA, USA). Qualitative data, including sex, fracture part, and presence of APR, were analyzed using Fisher’s Exact Test. Mean and standard deviation of continuous variables, such as age and BMD, were calculated and analyzed using Student’s *t*-test. Statistical significance was set at *p* < 0.05.

## Results

**Fig. 1:**
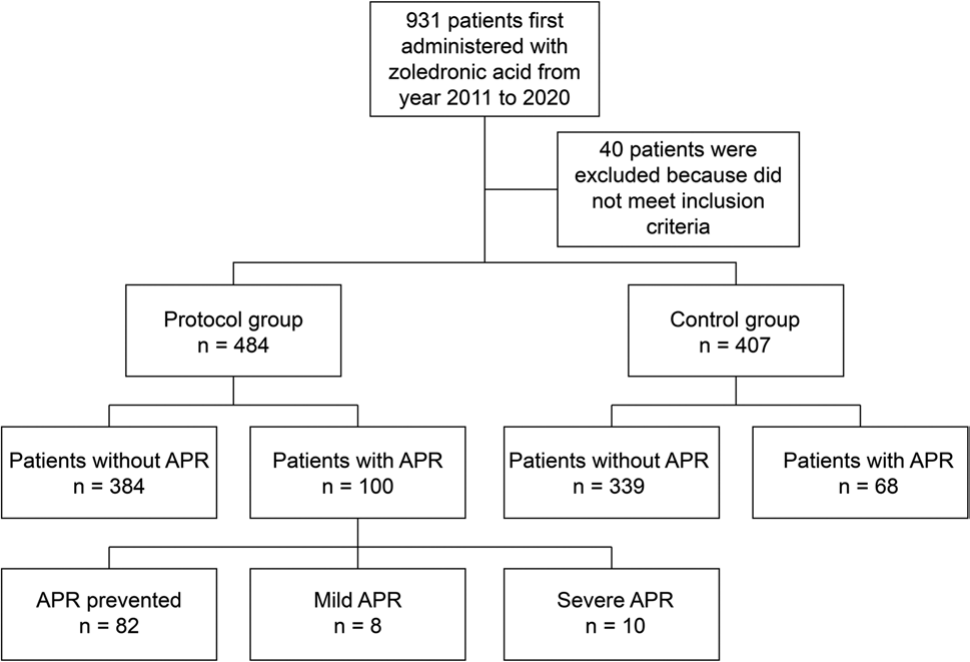
Workflow of participant selection in the study APR: acute phase response

A total of 931 patients were included in the study, of which 40 were excluded from analysis as they did not meet the diagnostic criteria for osteoporosis (Fig. 1). The mean age of the protocol (*n* = 507) and control groups (*n* = 407) was 72.90 and 75.17, respectively, indicating that the control group was significantly older (Table 2). Both groups comprised more females than males; however, the control group had a significantly higher proportion of females. Osteoporotic fracture was recorded in 807 patients (90.5%). The high incidence may be attributed to the treatment criteria established by Taiwan’s National Health Insurance for zoledronic acid, which stipulate that only patients with low bone density (T ≤ –2.5) and at least one osteoporotic fracture of the spine or hip joint are eligible for treatment. APR occurred more frequently in younger participants (*p* < 0.0001), consistent with previous research [11].

**Table 2:** Baseline characteristics

Characteristics	Protocol group (*n* = 484)	Control group (*n* = 407)	*p*-value
Age	72.9 ± 8.9	75.17 ± 8.48	< 0.0001
Female, *n* (%)	345 (72.28)	360 (88.45)	< 0.0001
Osteoporotic fracture, *n* (%)	419 (86.21)	388 (95.33)	< 0.0001
Vertebral, *n* (%)	340 (70.25)	333 (81.82)	< 0.0001
Femoral, *n* (%)	117 (24.17)	64 (12.72)	0.0020
Radial, *n* (%)	12 (2.48)	1 (0.25)	0.0046
BMD			
Spine	–2.79 ± 1.12	–3.25 ± 1.12	< 0.0001
Femoral neck	–2.87 ± 0.73	–3.1 ± 0.73	< 0.0001
One-third radius	–3.30 ± 0.52	–3.5 ± 1.47	0.4672

**Fig. 2:**
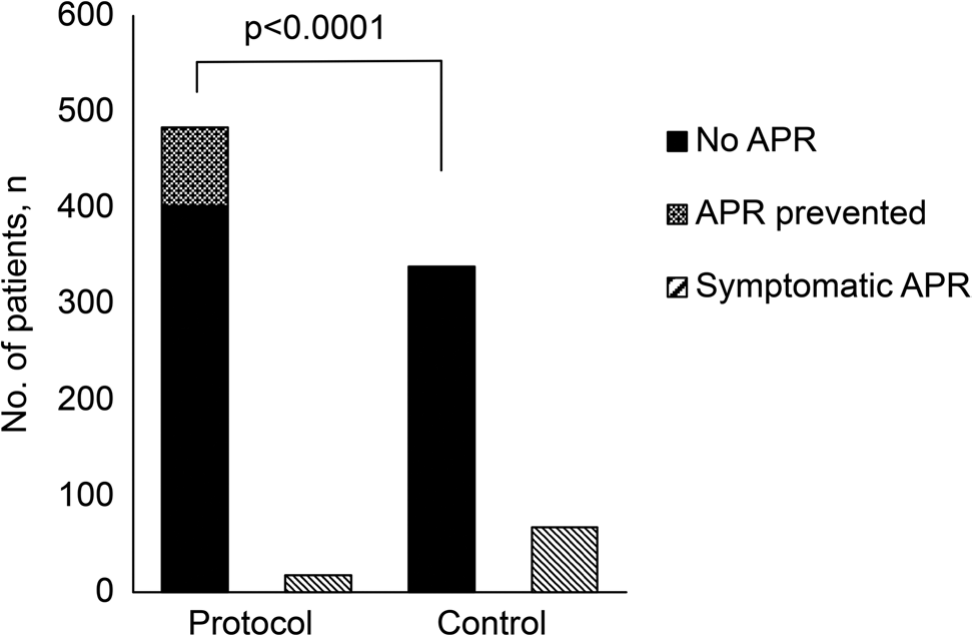
Comparison of APR incidence following zoledronic acid infusion between protocol and control groups

A total of 68 (16.71%) and 100 patients (20.66%) in the control and protocol group, respectively, reported symptoms of APR after zoledronic acid infusion; however, there was no difference between the two groups (*p* = 0.1442, Fisher’s exact test). In the protocol group, 82 patients (16.94%) showed complete relief from APR symptoms, 8 patients (1.65%) experienced partial relief, and only 10 patients (2.07%) had persistent APR symptoms. Overall, 466 out of 484 patients (96.28%) in the protocol group either experienced no APR symptoms or had tolerable symptoms, while 18 patients (3.72%) experienced intolerable symptoms. The APR remission rate was higher in the protocol group than that in the control group (*p* < 0.0001, Fisher’s exact test; Fig. 2).

The most common presentation of APR in both groups was diffuse musculoskeletal pain, affecting 36 (36.0%) and 46 patients (67.7%) in the protocol and control group, respectively. Fever was the second most observed symptom. Notably, diffuse musculoskeletal pain was also the most challenging symptom to control with medication, followed by general malaise (Table 3).

**Table 3:** Presentations of APR

Presentation	Protocol group (*n* = 100)		Control group (*n* = 68)		*p*-value
	**(*n*)**	**(%)**	**(*n*)**	**(%)**	
Fever	28	28.00%	23	33.82%	0.4947
Chillness	1	1.00%	2	2.94%	0.5664
Regional musculoskeletal pain	6	6.00%	0	0.00%	0.0822
Diffuse musculoskeletal	36	36.00%	46	67.65%	< 0.0001
Joint pain	1	1.00%	0	0.00%	> 0.9999
Nausea	0	0.00%	1	1.47%	0.4048
Vomiting	1	1.00%	1	1.47%	> 0.9999
Anorexia	2	2.00%	0	0.00%	0.5231
Eye inflammation	4	4.00%	0	0.00%	0.1479
Influenza-like illness	10	10.00%	0	0.00%	0.006
General malaise	19	19.00%	4	5.88%	0.0208
Dizziness	1	1.00%	0	0.00%	> 0.9999
Peripheral edema	1	1.00%	0	0.00%	> 0.9999
Only stated as APR	13	13.00%	0	0.00%	0.0009

*APR* acute phase response

## Discussion

Our findings indicated that while the combination of glucocorticoids, NSAIDs, and acetaminophen did not significantly reduce the overall incidence of zoledronic acid-induced APR, it was effective in reducing the severity of APR symptoms in patients. The incidence of symptomatic APR remained at 3.72%, suggesting that the primary benefit of this protocol lies in minimizing the intensity of symptoms. This highlights the importance of a comprehensive approach that includes both pre-infusion prophylaxis and post-exposure rescue therapy, to more effectively manage APR symptoms. As early as 1999, Kunzmann et al. elucidated the relationship between γ/δ T cell activity and APR, specifically that an increase in Vγ9Vδ2 T cells occurred in patients who developed APR [22]. Concurrently, Dicuonzo et al. showed that pro-inflammatory cytokines, such as tumor necrosis factor-alpha and interleukin-6, are key contributors to APR following zoledronic acid infusion [23]. Despite these insights, the molecular mechanism by which bisphosphonates stimulate the release of pro-inflammatory cytokines remains poorly understood [24]. However, it is believed that specific pro-inflammatory cytokines play an important role in triggering APR. Consequently, our combined protocol was developed to alleviate APR by inhibiting pro-inflammatory cytokine production both before and after zoledronic acid infusion.

In our prevention protocol, glucocorticoid is administered intravenously before infusion to attenuate pro-inflammatory cytokines. Upon binding to glucocorticoid receptors, glucocorticoid exerts an anti-inflammatory effect by transrepressing numerous pro-inflammatory genes, thereby inhibiting the synthesis of pro-inflammatory cytokines [25]. Hydrocortisone was selected due to its short-lived nature. Hydrocortisone administered alone intravenously demonstrated higher bioavailability and plasma concentration than a single dose of 4 mg oral dexamethasone [20, 26]. After zoledronic acid infusion, oral prednisolone was administered to prolong the treatment course and sustain glucocorticoid levels, accounting for hydrocortisone's short half-life [27]. Another rationale for selecting hydrocortisone and prednisolone over dexamethasone was that dexamethasone is typically reserved for acute or severe conditions [28].

In addition to glucocorticoids, NSAIDs were selected both for their anti-inflammatory effects as well as antipyretic and analgesic properties, which can alleviate the fever and pain associated with APR. Celecoxib was chosen due to its selective COX-2 inhibition, resulting in fewer gastrointestinal side effects compared to non-selective COX-1 inhibitors [29]. This mitigates the risk of patients confusing APR symptoms with medication-related gastrointestinal discomfort. Acetaminophen was incorporated in the protocol to provide additional symptom relief. Despite the unclear mechanism of acetaminophen, it is believed to act predominantly within the central nervous system, unlike NSAIDs which primarily act in the peripheral nervous system [30, 31]. Indeed, acetaminophen has shown great efficacy in reducing both the incidence and severity of APR [19].

Ocular inflammation is an uncommon presentation of APR induced by zoledronic acid [13]. Various ocular side effects, such as conjunctivitis, episcleritis, anterior uveitis (iritis), keratitis, and scleritis, have been reported [32–34]. While these adverse events are rare, the true incidence rate of ocular inflammation associated with zoledronic acid administration may be higher than previously estimated. For instance, Patel et al. reported an incidence rate of 0.8% for acute anterior uveitis in a prospective clinical trial, which represents "uncommon" incidence according to the WHO [35]. In our study, we observed an ocular inflammation incidence rate of 0.8% (4/484) in the protocol group following zoledronic acid infusion. The presentations varied from iritis to conjunctivitis, with recovery times ranging from days to months. Notably, no ocular inflammation events were reported in the control group. These findings highlight the potential risk of overlooking this severe side effect of zoledronic acid if thorough monitoring of all APR symptoms is not conducted, which could result in patient suffering and delayed treatment. It is imperative to inform patients about this potential adverse event when recommending zoledronic acid infusion. Moreover, when patients experience eye-related symptoms after injection, it is crucial that they be recommended an ophthalmologist for thorough diagnosis and appropriate treatment.

This study is limited by its retrospective cohort design, which introduces the potential for confirmation bias in the collected data. It is possible that initial APR symptoms may have been overlooked by some medical practitioners, while doctors in the protocol group were more vigilant in identifying and documenting all APR-related symptoms. Physicians using the combination protocol might have been more thorough in recording all instances of APR in the medical records, whereas those not using the protocol might have only documented more severe cases. In our study, the control group included only those APRs that required additional treatment due to severe symptoms, whereas the protocol group captured APRs ranging from mild to severe, potentially explaining the higher overall incidence of APRs. This could account for the higher rates of eye inflammation and the significantly greater occurrences of influenza-like illness and general malaise observed in the protocol group. In all cases of APR, this combined pharmacological treatment significantly reduced the incidence of symptomatic APR, particularly diffuse musculoskeletal pain. Although the results showed a higher rate of general malaise in the treatment group, most cases were mild and did not require any treatment. Only 5 patients in the treatment group required treatment for general malaise, which is comparable to the rate observed in the control group. To validate these findings, future research on combination therapy for preventing APR should be conducted prospectively. This approach would provide a more robust verification of the effectiveness of the prevention protocol.

## Conclusion

This study aimed to assess the efficacy of combination therapy in mitigating or managing APR triggered by zoledronic acid infusion in patients with osteoporosis. Our findings underscore the efficacy of the combination protocol comprising glucocorticoids, NSAIDs, and acetaminophen in alleviating zoledronic acid-induced APR. Future treatments involving zoledronic acid, especially for naïve patients, should integrate prevention strategies both before and after exposure to effectively alleviate APR discomfort. This approach could potentially improve patient compliance with scheduled doses, maximize treatment benefits, and improve patient outcomes.

## Data Availability

No data is available due to the ethical restrictions.

## References

[1] Johnell O, Kanis JA (2006) An estimate of the worldwide prevalence and disability associated with osteoporotic fractures. Osteoporos Int 17(12):1726–1733. 10.1007/s00198-006-0172-4 16983459 10.1007/s00198-006-0172-4

[2] Nazrun AS, Tzar MN, Mokhtar SA, Mohamed IN (2014) A systematic review of the outcomes of osteoporotic fracture patients after hospital discharge: morbidity, subsequent fractures, and mortality. Ther Clin Risk Manag 10:937–948. 10.2147/TCRM.S72456 25429224 10.2147/TCRM.S72456 PMC4242696

[3] MacLean C, Newberry S, Maglione M et al (2008) Systematic review: comparative effectiveness of treatments to prevent fractures in men and women with low bone density or osteoporosis. Ann Intern Med 148(3):197–213. 10.7326/0003-4819-148-3-200802050-00198 18087050 10.7326/0003-4819-148-3-200802050-00198

[4] Jeremiah MP, Unwin BK, Greenawald MH, Casiano VE (2015) Diagnosis and management of osteoporosis. Am Fam Physician 92(4):261–268 26280231

[5] Lippuner K, Pollock RF, Smith-Palmer J, Meury T, Valentine WJ (2011) A review of the cost effectiveness of bisphosphonates in the treatment of post-menopausal osteoporosis in Switzerland. Appl Health Econ Health Policy 9(6):403–417. 10.2165/11592210-000000000-00000 21910511 10.2165/11592210-000000000-00000

[6] Black DM, Delmas PD, Eastell R et al (2007) Once-yearly zoledronic acid for treatment of postmenopausal osteoporosis. N Engl J Med 356(18):1809–1822. 10.1056/NEJMoa067312 17476007 10.1056/NEJMoa067312

[7] Reid IR, Brown JP, Burckhardt P et al (2002) Intravenous zoledronic acid in postmenopausal women with low bone mineral density. N Engl J Med 346(9):653–661. 10.1056/NEJMoa011807 11870242 10.1056/NEJMoa011807

[8] Liu Z, Li CW, Mao YF et al (2019) Study on zoledronic acid reducing acute bone loss and fracture rates in elderly postoperative patients with intertrochanteric fractures. Orthop Surg 11(3):380–385. 10.1111/os.12460 31058448 10.1111/os.12460 PMC6595103

[9] Saag K, Lindsay R, Kriegman A, Beamer E, Zhou W (2007) A single zoledronic acid infusion reduces bone resorption markers more rapidly than weekly oral alendronate in postmenopausal women with low bone mineral density. Bone 40(5):1238–1243. 10.1016/j.bone.2007.01.016 17347063 10.1016/j.bone.2007.01.016

[10] McClung MR, Wagman RB, Miller PD, Wang A, Lewiecki EM (2017) Observations following discontinuation of long-term denosumab therapy. Osteoporos Int 28(5):1723–1732. 10.1007/s00198-017-3919-1 28144701 10.1007/s00198-017-3919-1 PMC5391373

[11] Cummings SR, Ferrari S, Eastell R et al (2018) Vertebral fractures after discontinuation of denosumab: a post hoc analysis of the randomized placebo-controlled FREEDOM trial and its extension. J Bone Miner Res 33(2):190–198. 10.1002/jbmr.3337 29105841 10.1002/jbmr.3337

[12] Spangeus A, Johansson S, Woisetschlager M (2020) Adherence to and persistence with zoledronic acid treatment for osteoporosis-reasons for early discontinuation. Arch Osteoporos 15(1):58. 10.1007/s11657-020-00733-4 32303862 10.1007/s11657-020-00733-4 PMC7165128

[13] Reid IR, Gamble GD, Mesenbrink P, Lakatos P, Black DM (2010) Characterization of and risk factors for the acute-phase response after zoledronic acid. J Clin Endocrinol Metab 95(9):4380–4387. 10.1210/jc.2010-0597 20554708 10.1210/jc.2010-0597

[14] Adami S, Bhalla AK, Dorizzi R et al (1987) The acute-phase response after bisphosphonate administration. Calcif Tissue Int 41(6):326–331. 10.1007/BF02556671 3124942 10.1007/BF02556671

[15] Welton JL, Morgan MP, Marti S et al (2013) Monocytes and γδ T cells control the acute-phase response to intravenous zoledronate: insights from a phase IV safety trial. J Bone Miner Res 28(3):464–471. 10.1002/jbmr.1797 23074158 10.1002/jbmr.1797

[16] Crotti C, Watts NB, De Santis M et al (2018) Acute phase reactions after zoledronic acid infusion: protective role of 25-hydroxyvitamin D and previous oral bisphosphonate therapy. Endocr Pract 24(5):405–410. 10.4158/EP161638.OR 29498910 10.4158/EP161638.OR

[17] Wei J, Zhu A, Ji JS (2019) A comparison study of vitamin D deficiency among older adults in China and the United States. Sci Rep 9(1):19713. 10.1038/s41598-019-56297-y 31873182 10.1038/s41598-019-56297-y PMC6928152

[18] Cauley JA, Danielson ME, Boudreau R et al (2011) Serum 25-hydroxyvitamin D and clinical fracture risk in a multiethnic cohort of women: the Women’s Health Initiative (WHI). J Bone Miner Res 26(10):2378–2388. 10.1002/jbmr.449 21710614 10.1002/jbmr.449 PMC3304434

[19] Silverman SL, Kriegman A, Goncalves J, Kianifard F, Carlson T, Leary E (2011) Effect of acetaminophen and fluvastatin on post-dose symptoms following infusion of zoledronic acid. Osteoporos Int 22(8):2337–2345. 10.1007/s00198-010-1448-2 21116816 10.1007/s00198-010-1448-2 PMC3132314

[20] Billington EO, Horne A, Gamble GD, Maslowski K, House M, Reid IR (2017) Effect of single-dose dexamethasone on acute phase response following zoledronic acid: a randomized controlled trial. Osteoporos Int 28(6):1867–1874. 10.1007/s00198-017-3960-0 28233020 10.1007/s00198-017-3960-0

[21] Okimoto N, Sakai A, Yoshioka T et al (2020) Efficacy of non-steroidal anti-inflammatory drugs on zoledronic acid-induced acute-phase reactions: randomized, open-label, Japanese OZ study. J Bone Miner Metab 38(2):230–239. 10.1007/s00774-019-01050-8 31586241 10.1007/s00774-019-01050-8

[22] Kunzmann V, Bauer E, Wilhelm M (1999) γ/δ T-cell stimulation by pamidronate. N Engl J Med 340(9):737–738. 10.1056/NEJM199903043400914 10068336 10.1056/NEJM199903043400914

[23] Dicuonzo G, Vincenzi B, Santini D et al (2003) Fever after zoledronic acid administration is due to increase in TNF-α and IL-6. J Interferon Cytokine Res 23(11):649–654. 10.1089/107999003322558782 14651779 10.1089/107999003322558782

[24] Thompson K, Roelofs AJ, Jauhiainen M, Monkkonen H, Monkkonen J, Rogers MJ (2010) Activation of γδ T cells by bisphosphonates. Adv Exp Med Biol 658:11–20. 10.1007/978-1-4419-1050-9_2 19950011 10.1007/978-1-4419-1050-9_2

[25] Vandevyver S, Dejager L, Tuckermann J, Libert C (2013) New insights into the anti-inflammatory mechanisms of glucocorticoids: an emerging role for glucocorticoid-receptor-mediated transactivation. Endocrinology 154(3):993–1007. 10.1210/en.2012-2045 23384835 10.1210/en.2012-2045

[26] Spoorenberg SM, Deneer VH, Grutters JC et al (2014) Pharmacokinetics of oral vs. intravenous dexamethasone in patients hospitalized with community-acquired pneumonia. Br J Clin Pharmacol 78(1):78–83. 10.1111/bcp.12295 24400953 10.1111/bcp.12295 PMC4168382

[27] Czock D, Keller F, Rasche FM, Haussler U (2005) Pharmacokinetics and pharmacodynamics of systemically administered glucocorticoids. Clin Pharmacokinet 44(1):61–98. 10.2165/00003088-200544010-00003 15634032 10.2165/00003088-200544010-00003

[28] Liu D, Ahmet A, Ward L et al (2013) A practical guide to the monitoring and management of the complications of systemic corticosteroid therapy. Allergy Asthma Clin Immunol 9(1):30. 10.1186/1710-1492-9-30 23947590 10.1186/1710-1492-9-30 PMC3765115

[29] Bacchi S, Palumbo P, Sponta A, Coppolino MF (2012) Clinical pharmacology of non-steroidal anti-inflammatory drugs: a review. Antiinflamm Antiallergy Agents Med Chem 11(1):52–64. 10.2174/187152312803476255 22934743 10.2174/187152312803476255

[30] Anderson BJ (2008) Paracetamol (Acetaminophen): mechanisms of action. Paediatr Anaesth 18(10):915–921. 10.1111/j.1460-9592.2008.02764.x 18811827 10.1111/j.1460-9592.2008.02764.x

[31] Smith HS (2009) Potential analgesic mechanisms of acetaminophen. Pain Physician 12(1):269–280 19165309

[32] McKague M, Jorgenson D, Buxton KA (2010) Ocular side effects of bisphosphonates: a case report and literature review. Can Fam Physician 56(10):1015–1017 20944044 PMC2954081

[33] Anandasayanan K, Malaravan M, Suganthan N (2020) Acute unilateral anterior uveitis following zoledronic acid infusion: a case report. SAGE Open Med Case Rep 8. 10.1177/2050313X20944305 10.1177/2050313X20944305 PMC7375717 32742658

[34] Benderson D, Karakunnel J, Kathuria S, Badros A (2006) Scleritis complicating zoledronic acid infusion. Clin Lymphoma Myeloma 7(2):145–147. 10.3816/CLM.2006.n.053 17026827 10.3816/CLM.2006.n.053

[35] Patel DV, Horne A, House M, Reid IR, McGhee CN (2013) The incidence of acute anterior uveitis after intravenous zoledronate. Ophthalmology 120(4):773–776. 10.1016/j.ophtha.2012.10.028 23290982 10.1016/j.ophtha.2012.10.028

